# Association of GLP‐1 Receptor Agonists With Risk of Suicidal Ideation and Behaviour: A Systematic Review and Meta‐Analysis

**DOI:** 10.1002/dmrr.70037

**Published:** 2025-02-13

**Authors:** Ganesh Bushi, Mahalaqua Nazli Khatib, Shivam Rohilla, Mahendra Pratap Singh, Nidhi Uniyal, Suhas Ballal, Pooja Bansal, Kiran Bhopte, Manika Gupta, Abhay M. Gaidhane, Balvir S. Tomar, Ayash Ashraf, M. Ravi Kumar, Ashish Singh Chauhan, Sanjit Sah, Hashem Abu Serhan, Muhammed Shabil

**Affiliations:** ^1^ School of Pharmaceutical Sciences Lovely Professional University Phagwara India; ^2^ Division of Evidence Synthesis Global Consortium of Public Health and Research Datta Meghe Institute of Higher Education Wardha India; ^3^ Department of Pharmacy Practice National Institute of Pharmaceutical Education and Research Guwahati India; ^4^ Evidence for Policy and Learning Global Center for Evidence Synthesis Chandigarh India; ^5^ Center for Global Health Research Saveetha Medical College and Hospital Saveetha Institute of Medical and Technical Sciences Saveetha University Chennai India; ^6^ Department of General Medicine Graphic Era (Deemed to be University) Dehradun India; ^7^ Department of Chemistry and Biochemistry School of Sciences JAIN (Deemed to be University) Bangalore India; ^8^ Department of Allied Healthcare and Sciences Vivekananda Global University Jaipur India; ^9^ IES Institute of Pharmacy IES University Bhopal India; ^10^ New Delhi Institute of Management Delhi India; ^11^ School of Epidemiology and Public Health Jawaharlal Nehru Medical College, and Global Health Academy Datta Meghe Institute of Higher Education Wardha India; ^12^ Institute of Pediatric Gastroenterology and Hepatology NIMS University Jaipur India; ^13^ Chandigarh Pharmacy College Chandigarh Group of College Mohali India; ^14^ Department of Chemistry Raghu Engineering College Visakhapatnam India; ^15^ Division of Research and Innovation Uttaranchal Institute of Pharmaceutical Sciences Uttaranchal University Dehradun India; ^16^ Department of Paediatrics Dr. D. Y. Patil Medical College Hospital and Research Centre Dr. D. Y. Patil Vidyapeeth Pune India; ^17^ Department of Public Health Dentistry Dr. D.Y. Patil Dental College and Hospital Dr. D.Y. Patil Vidyapeeth Pune India; ^18^ Department of Ophthalmology Hamad Medical Corporation Doha Qatar; ^19^ University Center for Research and Development Chandigarh University Mohali India; ^20^ Medical Laboratories Techniques Department AL‐Mustaqbal University Hillah Iraq

**Keywords:** diabetes, GLP‐1 receptor agonists, meta‐analysis, suicidal ideation

## Abstract

**Background and Objective:**

Glucagon‐like peptide‐1 receptor agonists (GLP‐1RAs) are widely used to treat type 2 diabetes and obesity, providing metabolic and cardiovascular benefits. However, concerns have emerged about potential neuropsychiatric side effects, including suicidal ideation and behaviour, prompting investigations by regulatory bodies such as the FDA and EMA. This systematic review and meta‐analysis aimed to assess the association between GLP‐1RA use and the risk of suicidal ideation or behaviour.

**Methods:**

A systematic literature search was conducted in PubMed, Embase, and Web of Science through September 2024, adhering to PRISMA guidelines. Observational cohort and case‐control studies reporting suicidal ideation or behaviour in adults using GLP‐1RAs were included. The Modified Newcastle‐Ottawa Scale assessed risk of bias, and random‐effect models calculated risk ratios (RR) with 95% confidence intervals (CIs). Heterogeneity was assessed using the I^2^ statistic.

**Results:**

Of 126 studies, 11 were included from multiple countries with diverse designs. The meta‐analysis of four studies showed no statistically significant difference in suicidal outcomes between GLP‐1RA users and users of other anti‐hyperglycaemic drugs (RR: 0.568, 95% CI: 0.077–4.205). Substantial heterogeneity was observed (I^2^ = 98%). Pharmacovigilance studies indicated no disproportionate increase in suicidality, while some observational studies suggested a lower risk.

**Conclusion:**

This review found no significant link between GLP‐1RA use and increased suicidal ideation or behaviour. However, the high heterogeneity and reliance on pharmacovigilance data suggest caution. Clinicians should monitor patients, particularly those with psychiatric conditions, and further research is needed to assess long‐term neuropsychiatric safety.

## 1 Introduction

Glucagon‐like peptide‐1 receptor agonists (GLP‐1RAs) are a class of medications primarily used to manage type 2 diabetes mellitus and obesity. These drugs have demonstrated favourable effects on glycaemic control, weight reduction, and cardiovascular outcomes, positioning them as pivotal components of modern diabetes treatment regimens [1, 2, 3]. However, recent post‐marketing surveillance has raised concerns about potential neuropsychiatric side effects, including suicidal ideation and behaviour, among patients using GLP‐1RAs. The United States Food and Drug Administration (FDA) and European Medicines Agency (EMA) have both issued safety communications and called for further investigation into this possible association [4].

While randomised controlled trials (RCTs) and observational studies have primarily focused on the metabolic and cardiovascular safety profiles of GLP‐1RAs, limited evidence exists regarding their effects on mental health outcomes [5]. Emerging data from clinical practice and pharmacovigilance databases have suggested that GLP‐1RAs might influence mood and behaviour, but findings remain inconclusive, and the magnitude of risk is not well‐defined [6]. Given the growing use of these agents and the seriousness of suicidal ideation as a potential adverse event, there is a critical need for a systematic evaluation of the available evidence to inform clinical decision‐making and regulatory guidelines.

This systematic review and meta‐analysis aimed to assess the association between GLP‐1RA use and the risk of suicidal ideation or behaviour by synthesising data from clinical trials and observational studies. By addressing this knowledge gap, we seek to clarify the potential neuropsychiatric risks associated with GLP‐1RAs and provide evidence‐based recommendations for clinicians and policymakers.

## 2 Methods

This systematic review and meta‐analysis were conducted following the PRISMA guidelines (Table S1) [7]. The study protocol was registered in the PROSPERO database.

### 2.1 Eligibility Criteria

Eligible studies included observational studies such as cohort studies and case‐control studies that examined the association between GLP‐1RA use and the risk of suicidal ideation or behaviour in adults. The inclusion criteria required studies to report on any neuropsychiatric outcomes, specifically focusing on suicidal ideation, suicide attempts, or completed suicides. Studies without control groups, studies involving paediatric populations, and non‐peer‐reviewed articles were excluded. There were no restrictions based on language or geographical location to ensure a comprehensive analysis of all relevant data.

### 2.2 Database Search

A comprehensive literature search was conducted using PubMed, Embase, and Web of Science for relevant articles from inception to September 8, 2024. Keywords and Medical Subject Headings (MeSH) terms related to GLP‐1RAs, suicidal ideation, and related neuropsychiatric outcomes were utilised. The search included both published and unpublished studies (e.g., conference abstracts). Additionally, a manual search of the references from the included studies and a grey literature search were performed to ensure comprehensive coverage of relevant data. A detailed search strategy is provided in Table S2.

### 2.3 Screening and Data Extraction

Nested Knowledge, a web‐based application, was utilised for screening and data extraction. Two independent reviewers screened the titles and abstracts of all identified studies, followed by full‐text review to confirm eligibility. Discrepancies were resolved through discussion or consultation with a third reviewer. Data extraction included study characteristics, participant demographics, follow‐up duration, and reported neuropsychiatric outcomes.

### 2.4 Quality Assessment

Risk of bias in the included studies was assessed using the Newcastle–Ottawa Scale for observational studies. This scale evaluates the quality of studies based on three main domains: selection of study groups, comparability of the groups, and ascertainment of outcomes. Studies were rated on these criteria, with higher scores indicating lower risk of bias. Discrepancies in scoring were resolved through discussion between the reviewers to ensure consistency.

### 2.5 Data Analysis

All statistical analyses were conducted using R version 4.4. The risk of suicidal ideation or behaviour was assessed using RR with 95% CIs. Heterogeneity across studies was evaluated using the I^2^ statistic, with values above 50% indicating substantial heterogeneity. A random‐effects model was applied to account for potential between‐study variability [8]. Statistical significance was set at a *p*‐value of < 0.05. If a minimum of 10 studies were available for meta‐analysis, publication bias was assessed using funnel plots and Egger's test [9, 10].

## 3 Results

### 3.1 Literature Search

**FIGURE 1:**
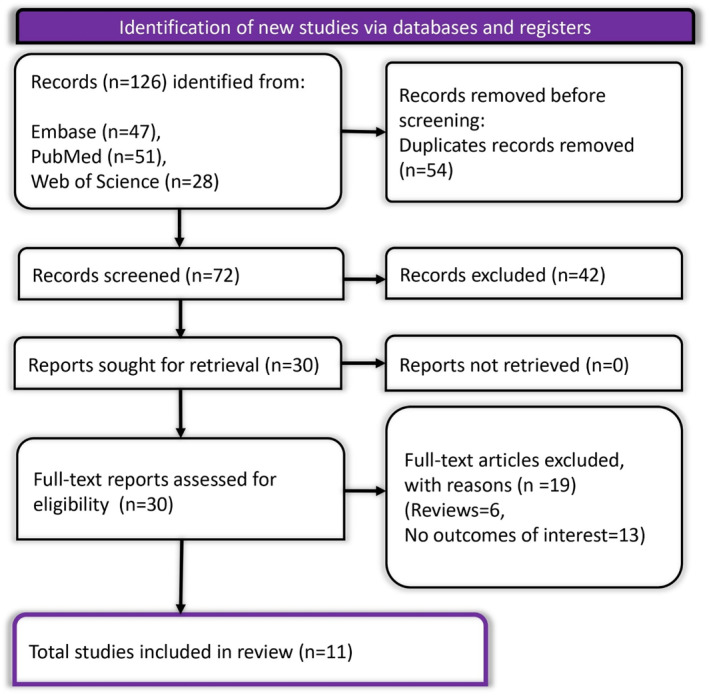
PRISMA flow diagram depicting the screening process.

A total of 126 records were identified through database searches (Embase: 47, PubMed: 51, Web of Science: 28). After removing 54 duplicates, 72 unique records were screened, of which 42 were excluded based on title and abstract review. Thirty full‐text articles were sought for retrieval, with none being unobtainable. After full‐text assessment, 19 articles were excluded: 6 were reviews and 13 did not report relevant outcomes. Ultimately, 11 studies met the inclusion criteria and were included in the final systematic review and meta‐analysis. The process is outlined in the PRISMA flow diagram (**Figure** **1**).

### 3.2 Basic Characteristics of Included Studies

The review included 11 studies from multiple countries, with 3 studies from the United States [11, 12, 13], 4 from multinationals [14, 15, 16, 17], 1 from China [18], 1 from Spain [19]. One from Italy [20], 1 from Saudi Arabia and Libya [21]. The studies employed diverse designs: 4 pharmacovigilance analyses [15, 16, 17, 18], 2 retrospective cohort studies [12, 13], one population‐based cohort study [19], one post hoc analysis [11], 1 case‐control study [14], 1 retrospective observational study [21], and 1 retrospective European pharmacovigilance study [20]. Various neuropsychiatric outcomes were assessed, including suicidal ideation, suicide attempts, suicidal behaviours, and self‐injury. Study populations ranged from type 2 diabetes patients, overweight/obese adults, and general users of GLP‐1RAs. The sample sizes of GLP‐1RA users varied widely from 357 to over 690,000 with suicide‐related events ranging from 4 to 534 across studies. Most studies utilised large pharmacovigilance databases such as the FDA Adverse Event Reporting System (FAERS) and the EudraVigilance database, with comparators including placebos, SGLT2 inhibitors, DPP‐4 inhibitors, and non‐GLP‐1RAs (**Table** **1**). The NOS revealed a moderate to high quality of the included studies (Table S3).

**TABLE 1:** Basic characteristics of included studies.

Study	Country	Study design	Age	Study population	Outcome	GLP‐1 sample size	Suicide events GLP‐1	Comparator	Comparator sample size	Suicide events comparator	Effect size
Chen 2023 [18]	China	Pharmacovigilance analysis	NA	GLP‐1RA‐associated suicide/self‐injury cases from FAERS (2005–2023)	Suicide/self‐injury	NA	534	NA	287,604	NA	ROR 0.16 (0.15–0.18) ‐ Indicates no disproportionate increase in suicidality with GLP‐1RAs
Guirguis 2024 [15]	Multi‐country	Descriptive and pharmacovigilance	NA	Adverse drug reports on GLP‐1RAs from FDA system (2005–2023)	Suicidal ideations	209,354 ADRs	383	NA	NA	NA	NA
Hurtado 2024 [19]	Spain	Population‐based cohort study	59 years	European ancestry in MR analysis from database (2015–2021)	Suicidal ideation and self‐injury	3040	4, 10	SGLT2i	11,627	25, 17	HR 1.04 (0.35, 3.14); HR 1.36 (0.51, 3.61) ‐ Indicates no significant increase in suicidal ideation with GLP‐1RA
Kim 2024 [16]	170 countries	Pharmacovigilance study	NA	Suicidality reports linked to GLP‐1RAs in global databases (2005–2023)	Suicidality	690,091	279	Other class of DM drugs	2,482,074	8595	No evidence of association; ROR 0.15 (0.13–0.16) for GLP‐1RAs ‐ Shows lower relative odds of suicidality
Nassar, Misra, and Bloomgarden 2024 [12]	United States	Retrospective cohort study	60.1, 65.7, 65.9 years	T2D patients from TriNetX network (data not specified, assumed current)	Suicidal attempt	373,041	106	DPP4	372,944	230	OR 0.461 (0.366, 0.58) ‐ Lower odds of suicidal attempts with GLP‐1RA compared to DPP4
O'Neil 2017 [11]	United States	Post hoc analysis	46.6 years	Overweight/obese adults enroled in a trial	NA	3270	34	PLACEBO	1832	19	NA
Ruggiero 2024 [20]	Italy	Retrospective European pharmacovigilance study	NA	Suicidal event reports linked to GLP‐1RAs from European database (2018–2023)	At least one suicidal event	41,236	230	NA	NA	NA	NA
Schoretsanitis 2024 [14]	Multinational	Case‐control study	48, 47 years	Reports of suicidal ADRs for liraglutide and semaglutide from WHO database (2000–2023)	All suicidal events	107, 162	50, 91	NA	NA	NA	NA
Tobaiqy 2024 [21]	Saudi Arabia, Libya	Retrospective observational study	49.8 years	Case safety reports from EudraVigilance database (2021–2023)	Suicide attempt, suicidal ideation	357	12, 69	NA	NA	NA	NA
Wang 2024 [13]	USA	Retrospective observational study	50.1, 57.5 years	Overweight or obesity patients using semaglutide	Suicide attempt, intentional self‐injury	865	6.4%, 4.7%	Non‐GLP1R agonists	865	5%, 3.7%	HR 0.27 (0.20, 0.36), HR 0.44 (0.32, 0.60) ‐ Lower risk for suicidal ideation with semaglutide
Zhou 2024 [17]	Multicountry	Retrospective disproportionality analysis	NA	Suicidal behaviours linked to GLP‐1RAs from FAERS (2018–2022)	Suicidal behaviours	91,431 cases	204	NA	NA	NA	NA

### 3.3 Narrative Synthesis

The systematic review included 11 studies from various countries employing different designs, including pharmacovigilance analyses, cohort studies and observational analyses. Four studies were based on pharmacovigilance data from large adverse event reporting systems such as FAERS and global databases [15, 16, 17, 18]. These studies consistently reported no disproportionate increase in suicidality associated with GLP‐1RA use. Specifically, Chen 2023 and Kim 2024 demonstrated lower relative odds of suicidality with GLP‐1RAs compared to other diabetes medications, with relative odds ratios (ROR) of 0.16 and 0.15, respectively, indicating no heightened risk. Similarly, retrospective studies like Nassar 2024 found significantly lower odds of suicide attempts in GLP‐1RA users compared to DPP‐4 inhibitors, while Hurtado 2024, a population‐based cohort study, found no significant increase in suicidal ideation or self‐injury compared to SGLT2 inhibitors [12, 19].

Observational studies, such as Wang 2024, which focused on patients with obesity using semaglutide, reported a lower risk for suicidal ideation and self‐injury compared with non‐GLP‐1RAs, with hazard ratios of 0.27 and 0.44, suggesting a potential protective effect of GLP‐1Ras [13]. Similarly, studies utilising European and WHO pharmacovigilance databases (Ruggiero 2024, Schoretsanitis 2024) found no significant increase in suicidality with GLP‐1RA use [14, 20]. O'Neil 2017, a post hoc analysis of clinical trial data, also showed no substantial neuropsychiatric adverse events related to GLP‐1RA use in overweight and obese adults [11]. Overall, the majority of studies reported no increased risk of suicidality with GLP‐1RAs, and some even suggested a lower risk compared with other anti‐hyperglycaemic drugs or placebo.

### 3.4 Meta‐Analysis

**FIGURE 2:**
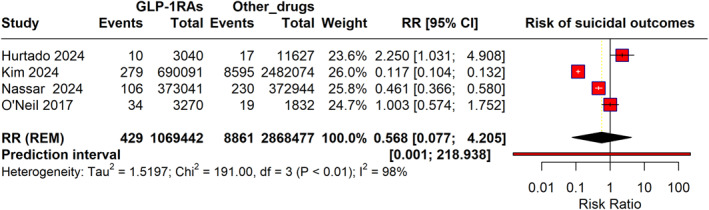
Forest plot illustrating the risk of suicidal outcomes in GLP‐1RAs.

Out of 11 studies, only 4 were eligible for meta‐analysis, pooling data to assess the risk of suicidal outcomes associated with GLP‐1RA compared with other anti‐hyperglycaemic drugs [11, 12, 16, 19]. The overall RR using a random‐effects model was 0.568 (95% CI: 0.077–4.205), indicating no statistically significant difference in the risk of suicidal outcomes between GLP‐1RAs and other drug classes. However, substantial heterogeneity was observed (I^2^ = 98%, *p* < 0.01), reflecting high variability across the included studies. The wide prediction interval (0.001–218.938) further suggests considerable uncertainty in the estimated effect (**Figure** **2**).

## 4 Discussion

The findings from this systematic review and meta‐analysis indicate that there is no statistically significant association between GLP‐1RA use and an increased risk of suicidal ideation or behaviour (RR: 0.568, 95% CI: 0.077–4.205). However, the high heterogeneity observed across studies (I^2^ = 98%) raises concerns about the reliability of this finding and necessitates cautious interpretation. The considerable heterogeneity likely stems from the diverse methodologies, populations, and comparators used in the included studies, which introduce variability in outcomes. Most of the included studies were based on pharmacovigilance databases, which are inherently prone to underreporting and reporting biases. These databases primarily capture spontaneous adverse event reports, which may not accurately reflect the true incidence of neuropsychiatric events such as suicidal ideation. Moreover, the wide prediction interval observed in the meta‐analysis further underscores the uncertainty surrounding the true relationship between GLP‐1RAs and suicidality. Given the seriousness of suicidal ideation as a clinical outcome, it remains imperative that clinicians maintain vigilance when prescribing GLP‐1RAs, especially in patients with pre‐existing psychiatric conditions who may be at elevated risk.

Despite these limitations, the results of this review are consistent with several recent studies that have explored the potential link between GLP‐1RA use and suicidality. For instance, Nguyen (2024) [12] conducted a Mendelian randomisation study that found no causal evidence linking the glycaemic and BMI‐lowering effects of GLP‐1RAs to an increased risk of suicide attempts, either in the general population or in individuals with pre‐existing mental health conditions. Nguyen's findings align with the results of RCTs of GLP‐1RAs, which have not demonstrated an increased risk of suicidality, although it is important to note that these trials typically included populations at lower risk for suicide, such as older adults or individuals without significant psychiatric histories. Additionally, real‐world evidence, particularly from propensity‐matched cohort studies, has suggested that semaglutide use may be associated with reductions in major depression and suicidal ideation. However, Nguyen et al. 2024 cautioned that small sample sizes limited these studies and were not adequately powered to exclude the possibility of suicide attempts.

Arillotta (2024) [22] employed a mixed‐method approach to analyse patient discussions about GLP‐1RAs on social media platforms, including Reddit, YouTube, and TikTok. Arillotta's analysis found mixed reports on mood and mental health changes after initiating GLP‐1RA therapy. While some users reported marked improvements in mood, better control of addictive behaviours, and reductions in anxiety, others reported increases in depressive symptoms, anxiety, and insomnia. These mixed perceptions highlight the complexity of patient experiences with GLP‐1RAs and underscore the difficulty of establishing a clear cause‐and‐effect relationship between these medications and suicidality based solely on patient reports. Nonetheless, the study offers valuable insight into how these drugs are perceived by the public and emphasises the need for further research to understand the full spectrum of neuropsychiatric effects associated with GLP‐1RAs.

Conversely, pharmacovigilance data have raised concerns about potential neuropsychiatric side effects, particularly with semaglutide. Schoretsanitis et al. (2024) [14] conducted a disproportionality analysis using the World Health Organization’s (WHO) global adverse drug reaction database, identifying 107 cases of suicidal and self‐injurious behaviours associated with semaglutide and 162 cases with liraglutide. The analysis found a significant signal for semaglutide‐related suicidal ideation, with a reporting odds ratio (ROR) of 1.45 (95% CI: 1.18–1.77). Notably, the risk was substantially higher in patients who were co‐prescribed antidepressants (ROR: 4.45, 95% CI: 2.52–7.86) or benzodiazepines (ROR: 4.07, 95% CI: 1.69–9.82), suggesting that individuals with pre‐existing mental health conditions may be more vulnerable to the neuropsychiatric effects of GLP‐1RAs. This finding is particularly important because it suggests a potential interaction between GLP‐1RAs and psychiatric medications, which may amplify the risk of adverse neuropsychiatric outcomes in certain populations. On the other hand, Guirguis et al. (2024) [15] analysed data from the FDA's FAERS database and found that among 5378 psychiatric adverse drug reactions (ADRs) related to GLP‐1RAs, there were 236 cases of suicidal ideation and 13 completed suicides across various GLP‐1RAs, including semaglutide and liraglutide. Although semaglutide had a relatively high ROR for suicidal ideation (2.03), no definitive causality could be established, and the overall incidence of suicide‐related events remained low.

Taken together, these studies highlight the complexity of assessing the association between GLP‐1RA use and suicidality. On the one hand, the available evidence from clinical trials, observational studies, and real‐world data largely suggests that GLP‐1RAs do not significantly increase the risk of suicidal ideation or behaviour [23]. In fact, some studies have even suggested a potential protective effect of these medications on mood and cognition, possibly due to the presence of GLP‐1 receptors in the central nervous system (CNS) [24]. GLP‐1 receptors are known to play a role in regulating appetite and energy homoeostasis, and their activation may indirectly influence mental health by modulating reward pathways, mood, and stress responses [25]. Furthermore, GLP‐1RAs are capable of crossing the blood‐brain barrier, and their neuroprotective effects may extend beyond metabolic regulation, potentially contributing to improvements in cognitive function and overall mental well‐being [26]. By mitigating metabolic disturbances such as obesity, diabetes, and associated neuroinflammation—conditions that frequently overlap with mental health disorders—GLP‐1RAs may provide secondary benefits for mental health [27].

However, the potential for neuropsychiatric side effects, particularly in vulnerable populations such as individuals with pre‐existing psychiatric conditions, cannot be ignored. The findings from pharmacovigilance databases, which report isolated cases of suicidal ideation and self‐injurious behaviour, particularly in patients co‐administered with psychiatric medications such as antidepressants or benzodiazepines, suggest that clinicians should be cautious when prescribing GLP‐1RAs to these populations. The potential for drug interactions and the heightened sensitivity of certain individuals to neuropsychiatric side effects warrant close monitoring of mental health in patients receiving GLP‐1RA therapy, particularly as these medications are increasingly prescribed for indications beyond diabetes management, including obesity and weight loss.

The strengths of the evidence included in this systematic review and meta‐analysis stem from the diversity of study designs, including observational cohort studies, and pharmacovigilance analyses. This wide range of data sources provides a comprehensive view of the potential neuropsychiatric effects of GLP‐1RAs. Additionally, the inclusion of real‐world data, such as studies utilising large pharmacovigilance databases such as FAERS and the WHO global database, allows for the identification of rare adverse events that may not be captured in clinical trials. Furthermore, the use of rigorous methodologies, such as adherence to PRISMA guidelines and the application of a random‐effects model in the meta‐analysis, enhances the robustness of the findings by accounting for between‐study variability.

There are notable limitations that affect the quality and reliability of the evidence. The substantial heterogeneity reported across the included studies (I^2^ = 98%) highlights the significant variability in study populations, methodologies, and comparators, which might impact the reliability of the pooled effect estimate. While the high level of heterogeneity suggests considerable variation in the study results, a more detailed discussion on the potential sources of this heterogeneity, such as differences in study design, population characteristics, and outcome definitions, would enhance the interpretability of the findings. Additionally, many of the included studies relied on pharmacovigilance data, which are inherently prone to underreporting and reporting biases, particularly for rare adverse events such as suicidal ideation. Furthermore, the exclusion of individuals with a history of mental illness from most clinical trials introduces selection bias, potentially underestimating the true risk of neuropsychiatric events in vulnerable populations. Finally, the relatively small number of studies included in the meta‐analysis, combined with wide prediction intervals, adds to the uncertainty regarding the true relationship between GLP‐1RA use and suicidal ideation or behaviour.

In terms of implications for clinical practice, policy, and future research, these findings underscore the importance of individualised patient care. While no strong evidence links GLP‐1RA use to an increased risk of suicidality, clinicians should exercise caution when prescribing these medications to patients with co‐existing psychiatric conditions. Close monitoring of mental health, particularly in individuals taking concurrent psychiatric medications, is warranted. The potential neuroprotective benefits of GLP‐1RAs, as suggested by some studies, highlight the need for further exploration of these drugs' effects on mood and cognition. For policy, regulatory bodies such as the FDA and EMA should continue to monitor post‐marketing safety data related to GLP‐1RAs, particularly as their use expands to broader populations for obesity and weight loss treatment. Updated clinical guidelines could include recommendations for screening patients with a history of psychiatric disorders before initiating GLP‐1RAs and advice on the importance of monitoring mental health during treatment.

Future research should focus on large‐scale, well‐designed longitudinal studies that include populations with psychiatric comorbidities to better understand the long‐term neuropsychiatric effects of GLP‐1RAs. Mechanistic studies investigating how GLP‐1RAs interact with the central nervous system (CNS) and their potential impact on mood and cognition will be crucial to further clarify their safety profile. Given the complexity of the relationship between metabolic and neuropsychiatric factors, future studies should also consider the potential influence of rapid weight loss, neuroinflammation, and metabolic improvement on mental health outcomes in patients using GLP‐1RAs.

## 5 Conclusion

This systematic review and meta‐analysis found no statistically significant association between GLP‐1RA use and increased risk of suicidal ideation or behaviour. However, substantial heterogeneity across studies and limitations inherent to pharmacovigilance data necessitate cautious interpretation. While most evidence suggests GLP‐1RAs do not elevate suicidality risk and may offer mood benefits, isolated reports of neuropsychiatric side effects, particularly in patients with pre‐existing psychiatric conditions, warrant continued monitoring. Clinicians should remain vigilant when prescribing GLP‐1RAs, especially for vulnerable populations, and further research is essential to clarify their long‐term neuropsychiatric safety.

## Author Contributions

Conceptualisation: S.R., B.S.T., A.M.G.; Data curation: M.G., M.S., H.A.S., S.S.; Formal analysis: A.S.C., A.A., M.G.; Investigation: G.B., A.S.C., K.B., S.R.; Methodology: P.B., S.B.; Project administration: S.R., G.B.; Resources: N.U., M.P.S., M.N.K.; Software: S.S., M.G., A.S.C.; Supervision: S.R., M.S.; Validation: G.B., H.A.S., S.S., M.R.K., A.A.; Visualisation: A.S.C., K.B.; Writing–original draft: S.R., N.U., P.B.; Writing–review & editing: G.B., S.S., S.R., M.G.

## Ethics Statement

The authors have nothing to report.

## Conflicts of Interest

The authors declare no conflicts of interest.

### Peer Review

The peer review history for this article is available at https://www.webofscience.com/api/gateway/wos/peer-review/10.1002/dmrr.70037.

## Supporting information

Table S1–S3

## Data Availability

The data are with the authors and available upon request.
